# Luteolin modulates SERCA2a via Sp1 upregulation to attenuate myocardial ischemia/reperfusion injury in mice

**DOI:** 10.1038/s41598-020-72325-8

**Published:** 2020-09-21

**Authors:** Ya Hu, Chengmeng Zhang, Hong Zhu, Shuai Wang, Yao Zhou, Jiaqi Zhao, Yong Xia, Dongye Li

**Affiliations:** 1 Institute of Cardiovascular Disease Research, Xuzhou Medical University, 84 West Huaihai Road, Xuzhou, 221002 Jiangsu People’s Republic of China; 2 Department of Cardiology, The Affiliated Hospital of Xuzhou Medical University, 99 West Huaihai Road, Xuzhou, 221002 Jiangsu People’s Republic of China

**Keywords:** Transcription, Carotid artery disease

## Abstract

The sarco/endoplasmic reticulum Ca^2+^ ATPase 2a (SERCA2a) is responsible for calcium transport during excitation–contraction coupling and is essential for maintaining myocardial systolic/diastolic function and intracellular Ca^2+^ levels. Therefore, it is important to investigate mechanisms whereby luteolin modulates SERCA2a expression to attenuate myocardial ischemia/reperfusion injury. C57BL/6j mice were randomly divided into eight groups. The expression and activity of SERCA2a was measured to assess interactions between the SERCA2a promoter and the Sp1 transcription factor, and the regulatory effects of luteolin. We used serum LDH release, serum cardiac troponin I level, hemodynamic data, myocardial infarction size and apoptosis-related indices to measure SERCA2a cardio-protective effects of luteolin pretreatment. Sp1 binding to SERCA2a promoter under ischemia/reperfusion conditions in the presence or absence of luteolin was analyzed by chromatin immunoprecipitation. Our experimental results indicated that during myocardial ischemia/reperfusion injury, luteolin pretreatment upregulated the expression levels of SERCA2a and Sp1. Sp1 overexpression enhanced the expression of SERCA2a at the transcriptional level. Luteolin pretreatment reversed the expression of SERCA2a through the increased expression of Sp1. Moreover, we demonstrated that luteolin pretreatment appeared to exert myocardial protective effects by upregulating the transcriptional activity of SERCA2a, via Sp1. In conclusion, during myocardial ischemia/reperfusion, Sp1 appeared to downregulate the expression of SERCA2a. Luteolin pretreatment was shown to improve SERCA2a expression via the upregulation of Sp1 to attenuate myocardial ischemia/reperfusion injury.

## Introduction

Due to improvements in living standards and associated rich diets, coronary heart disease (CHD) has become a major disease that threatens the health of many populations worldwide. At present, a series of coronary revascularizations can be implemented to treat CHD. However, in spite of localized recovery in the ischemic myocardium, these therapeutic methods also lead to myocardial ischemia/reperfusion (I/R) injury. Therefore, it is essential to determine mechanisms underlying this pathology and to verify the therapeutic effects of novel medicines that attenuate myocardial I/R injury.

Several studies have described the pathological process of myocardial I/R injury^1–3^. The injury appears to involve a variety of mechanisms, including calcium overload, oxidative stress and mitochondrial damage which triggers myocardial apoptosis and necrosis. Accordingly, abnormal intracellular calcium concentrations are associated with the myocardial I/R injury^4–7^. Sarco/endoplasmic reticulum Ca^2+^ ATPase 2a (SERCA2a) is responsible for transporting calcium from the cytosol into the sarcoplasmic reticulum (SR) during excitation–contraction coupling. The protein is essential in maintaining myocardial systolic/diastolic function and intracellular Ca^2+^ levels, therefore SERCA2a has been proposed to play key roles in the cardiac cycle.

At present, SERCA2a is closely associated with I/R injury^8,9^; in their study, Netticadan. et al*.,* suggested that SERCA2a phosphorylation was downregulated during I/R injury^10^. It is now well-established that this downregulation of SERCA2a phosphorylation and SERCA2a promoter activity are involved in myocardial I/R injury^11–13^. Our laboratory observed the downregulation of SERCA2a expression and SERCA2a mRNA levels when rats and mice were subjected to myocardial I/R injury^14,15^. It was also demonstrated that myocardial cells exhibited a reduced Ca^2+^ transporting capability, from the cytosol into the SR, when SERCA2a was downregulated in reperfused cardiomyocytes. Aberrant, high cytoplasmic Ca^2+^ levels ultimately lead to cardiomyocyte apoptosis or necrosis^16^.

The transcription factor specific protein 1 (Sp1), which is a sequence specific DNA binding protein, belongs to a subfamily of zinc finger proteins^17^. Its carboxyl terminus is highly conserved and contains three Cys_2_His_2_ zinc finger structures, which bind directly to the GC box of chromosomal DNA^18,19^. The *cis*-acting element of the SERCA2a 5′ proximal region is GC rich, and its promoter region contains Sp1 binding sites^20^. Therefore, it is of great importance to explore interactions between promoters and transcription factors that modulate gene expression, implicated in disease mechanisms.

Luteolin (Lut), is a natural soluble flavone, present in fruit and vegetables, with multiple pharmacological activities^21,22^. Lut has been shown to activate the endothelial nitric oxide synthase (eNOS) pathway, enhance superoxide dismutase function and weaken mitochondrial permeability transition pore function in cardiomyocytes^23^. To date, no large-scale studies have explored Lut mechanisms, which protect myocardium from reperfusion injury.

Takizawa et al*.,* observed that mutation of Sp1 sites I (− 196 to − 191 bp) and III (− 118 to − 13 bp) in pressure-loaded rats, downregulated the transcriptional activity of SERCA2a, and blocked the inhibitory action of pressure overload, when compared with sham groups^20^. Palbykin et al*.,* demonstrated that trichloroethylene up-regulated SERCA2a transcriptional activity, enhanced the binding of Sp1 and the SERCA2a promoter region through the induction of SERCA2a methylation in H9c2 cells^24^.

Notwithstanding these observations, the literature has not provided robust evidence on whether Sp1 participates in the modulation of SERCA2a during myocardial I/R injury. Thus, the purpose of the present study was to assess whether or not Lut regulated SERCA2a via Sp1, in order to protect mouse myocardium tissue from I/R injury. In performing these studies, we provide theoretical and experimental evidence for future clinical applications.

## Results

### Lut administration increases the expression and transcription of Sp1 and SERCA2a

**Figure 1:**
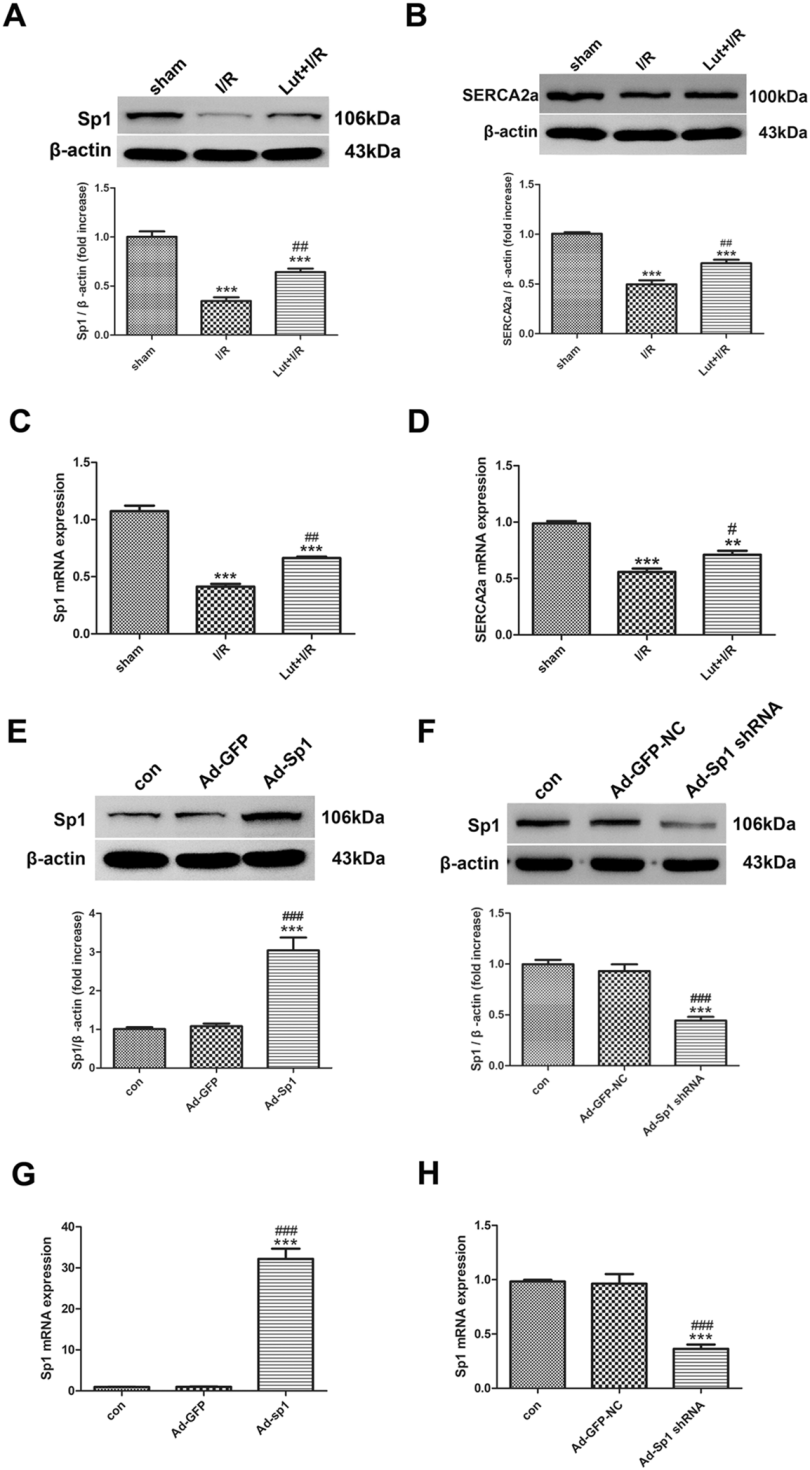
Lut increased the expression and transcription of Sp1 and SERCA2a in vivo. The efficiency of Sp1 overexpression and Sp1 knockdown. **(A)** Expression of Sp1 by different treatments. **(B)** Expression of SERCA2a in each treatment group. **(C)** Expression of Sp1 mRNA by different treatments. **(D)** Expression of SERCA2a mRNA in each treatment group. **(E, F)** Expression of Sp1 in each treatment group. **(G, H)** Expression of Sp1 mRNA in each treatment group. Data are represented as mean ± SEM (n = 4). The full-length blots are presented in Supplemental Fig. 2. ^**^P < 0.01, ^***^P < 0.001 versus sham; ^#^*P* < 0.05, ^##^*P* < 0.01, ^###^*P* < 0.001 versus I/R.

As shown (Fig. 1A,B), the expression of Sp1 and SERCA2a was downregulated in the I/R group, in comparison with the sham group (*P* < 0.001, respectively). However, these expression levels were partly restored by Lut (*P* < 0.01, respectively).

Furthermore, the tendency of Sp1 and SERCA2a mRNA levels in the above three groups agreed with the tendency of protein expression levels in vivo (Fig. 1C,D).

### Sp1 overexpression and Sp1 knockdown efficiencies

The efficiency of Sp1 overexpression and Sp1 knockdown are shown (Fig. 1E–H). Sp1 level and mRNA expression were significantly upregulated in myocardial tissue transfected by the Sp1 overexpression adenovirus (*P* < 0.001). Moreover, after a 3-day transfection with the Sp1 knockdown adenovirus, Sp1 expression was reduced to less than 40%, when compared with expression in the control group of strain-matched WTs (*P* < 0.001). However, Sp1 levels in the Ad-GFP and Ad-GFP-NC groups, which were transfected with Sp1 overexpression control adenovirus or Sp1 knockdown control adenovirus, were not significantly altered, when compared with the control group.

### Lut administration increases the expression and transcription of SERCA2a and enhances SERCA2a activity via the upregulation of Sp1

**Figure 2:**
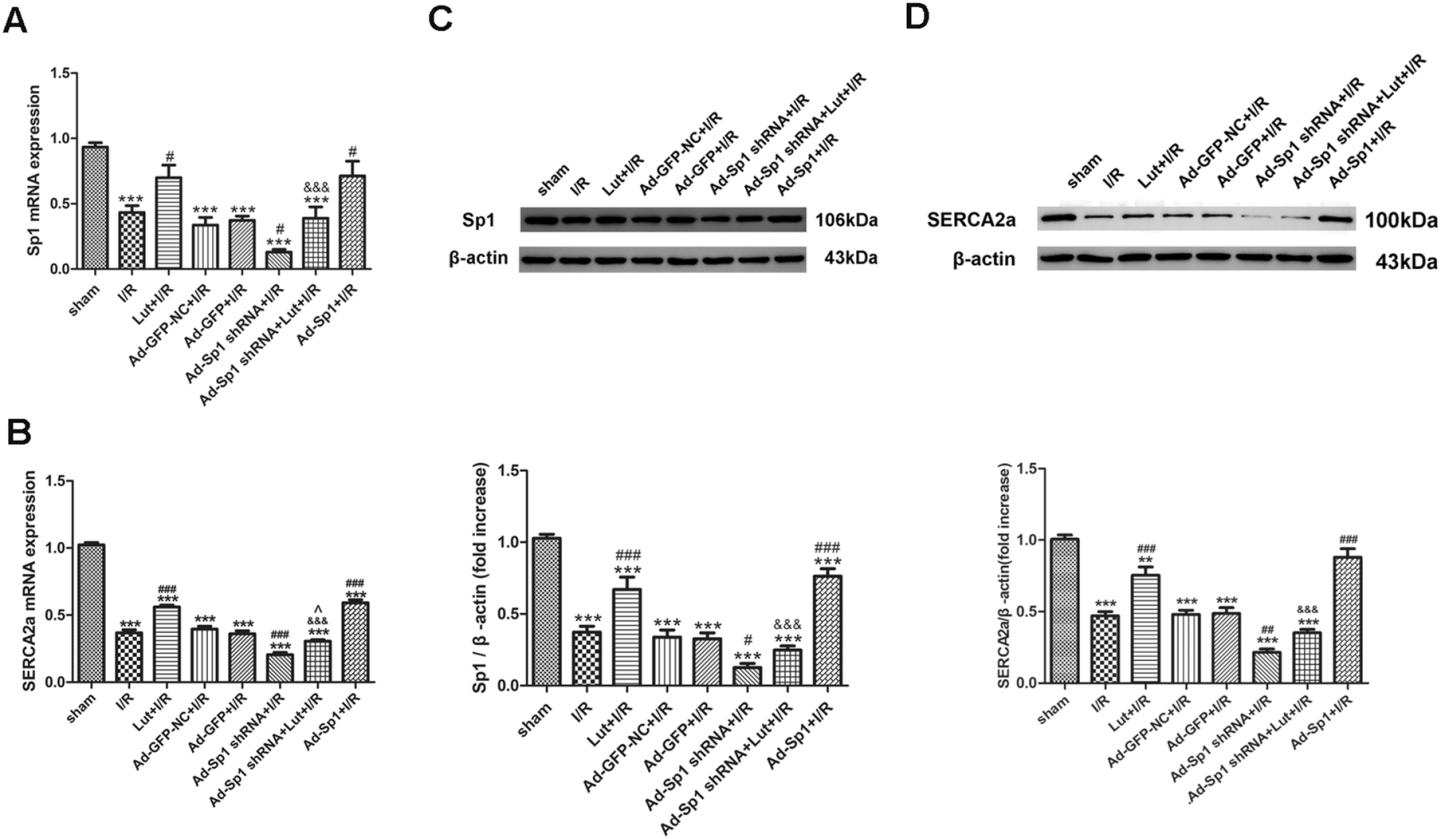
Luteolin increased the expression and transcription of SERCA2a via the upregulation of Sp1. **(A)** Expression of Sp1 mRNA in each treatment group. **(B)** Expression of SERCA2a mRNA in each treatment group. **(C)** The level of Sp1 expression in each treatment group. **(D)** The level of SERCA2a expression in each treatment group. Data are represented as mean ± SEM (n = 3–5). The full-length blots are presented in Supplemental Fig. 3. ^***^P < 0.001 versus sham; ^#^*P* < 0.05, ^##^*P* < 0.01, ^###^*P* < 0.001 versus I/R; ^&&&^*P* < 0.001 versus Lut + I/R; ^^^*P* < 0.05 versus Ad-Sp1 shRNA + I/R.

Sp1 and SERCA2a mRNA expression levels in the experimental groups were measured by RT-PCR (Fig. 2A,B). Sp1 was overexpressed or knocked down to investigate the relationship between SERCA2a and Sp1. Compared with the sham group, Sp1 and SERCA2a mRNA levels in the I/R group were markedly downregulated in comparison with the sham group (*P* < 0.001). However, levels were lower than those in the Lut + I/R group, suggesting that Lut pretreatment appeared to partly reverse Sp1 and SERCA2a mRNA expression levels (*P* < 0.05, *P* < 0.001, respectively).

After transfection with Sp1 overexpression control adenovirus or Sp1 knockdown control adenovirus, compared with the I/R group, there were no significant differences in Sp1 and SERCA2a mRNA expression in the Ad-GFP-NC + I/R group and Ad-GFP + I/R group (Fig. 2A,B).

When Sp1 was knocked down, Sp1 and SERCA2a mRNA expression levels in the Ad-Sp1 shRNA + I/R group were downregulated, when compared with the I/R group (*P* < 0.05, *P* < 0.001, respectively). Sp1 and SERCA2a mRNA expression levels were significantly upregulated once Sp1 was overexpressed in the Ad-Sp1 + I/R group, during I/R injury (*P* < 0.05, *P* < 0.001, respectively). Furthermore, Sp1 and SERCA2a mRNA expression in the Ad-Sp1 shRNA + Lut + I/R group was significantly lower than that in the Lut + I/R group (*P* < 0.001). In addition, SERCA2a mRNA expression in the Ad-Sp1 shRNA + Lut + I/R group was upregulated, in comparison to expression in the Ad-Sp1 shRNA + I/R group (*P* < 0.05) (Fig. 2A,B).

**Figure 3:**
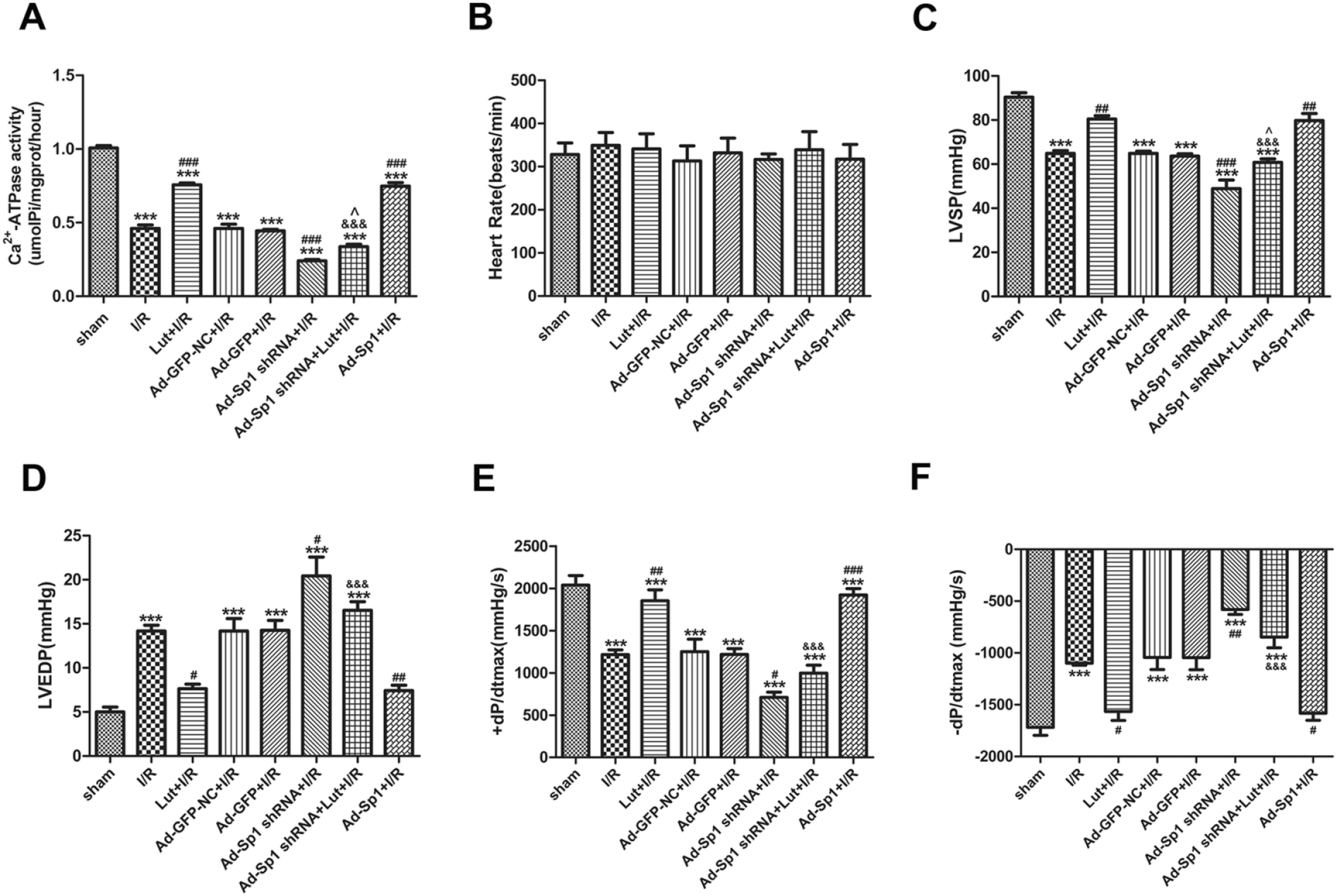
Lut enhances SERCA2a activity via the upregulation of Sp1 and modulates SERCA2a via Sp1 to enhance systolic/diastolic function of the myocardium. **(A)** Change of SERCA2a activity in each treatment group. **(B–F)** Effects of different treatments on the myocardial hemodynamic indices. Data are represented as mean ± SEM (n = 4–5). ^***^P < 0.001 versus sham; ^#^*P* < 0.05, ^##^*P* < 0.01, ^###^*P* < 0.001 versus I/R; ^&&&^*P* < 0.001 versus Lut + I/R; ^^^*P* < 0.05 versus Ad-Sp1 shRNA + I/R.

Sp1 and SERCA2a expression were correlated to the mRNA expression trends in the different groups, as well as the SERCA2a activity (Figs. 2C, D, 3A).

### Lut modulates SERCA2a via the upregulation of Sp1 to enhance systolic/diastolic function of the myocardium

There were no differences in heart rates among all groups tested (Fig. 3B). As shown (Fig. 3C–F), a significant increase in LVEDP appeared in the I/R group, when compared with the sham group, as well as reductions in LVSP, + dP/dtmax and -dP/dtmax in the I/R group (*P* < 0.001). In addition, Lut pretreatment partly reversed variations of these indices in the I/R group (*P* < 0.05, *P* < 0.01, respectively).

LVEDP was increased in the Ad-Sp1 shRNA + I/R group and decreased in the Ad-Sp1 + I/R group, in comparison to the I/R group (*P* < 0.05, *P* < 0.01, respectively). When compared to the Lut + I/R group, LVEDP in the Ad-Sp1 shRNA + Lut + I/R group was up-regulated (*P* < 0.001). However, LVSP, + dP/dt max and − dP/dt max in the Ad-Sp1 shRNA + Lut + I/R group were all decreased (Fig. 3C–F).

### Lut modulates SERCA2a to decrease serum LDH release and cardiac troponin I level via the up-regulation of Sp1

**Figure 4:**
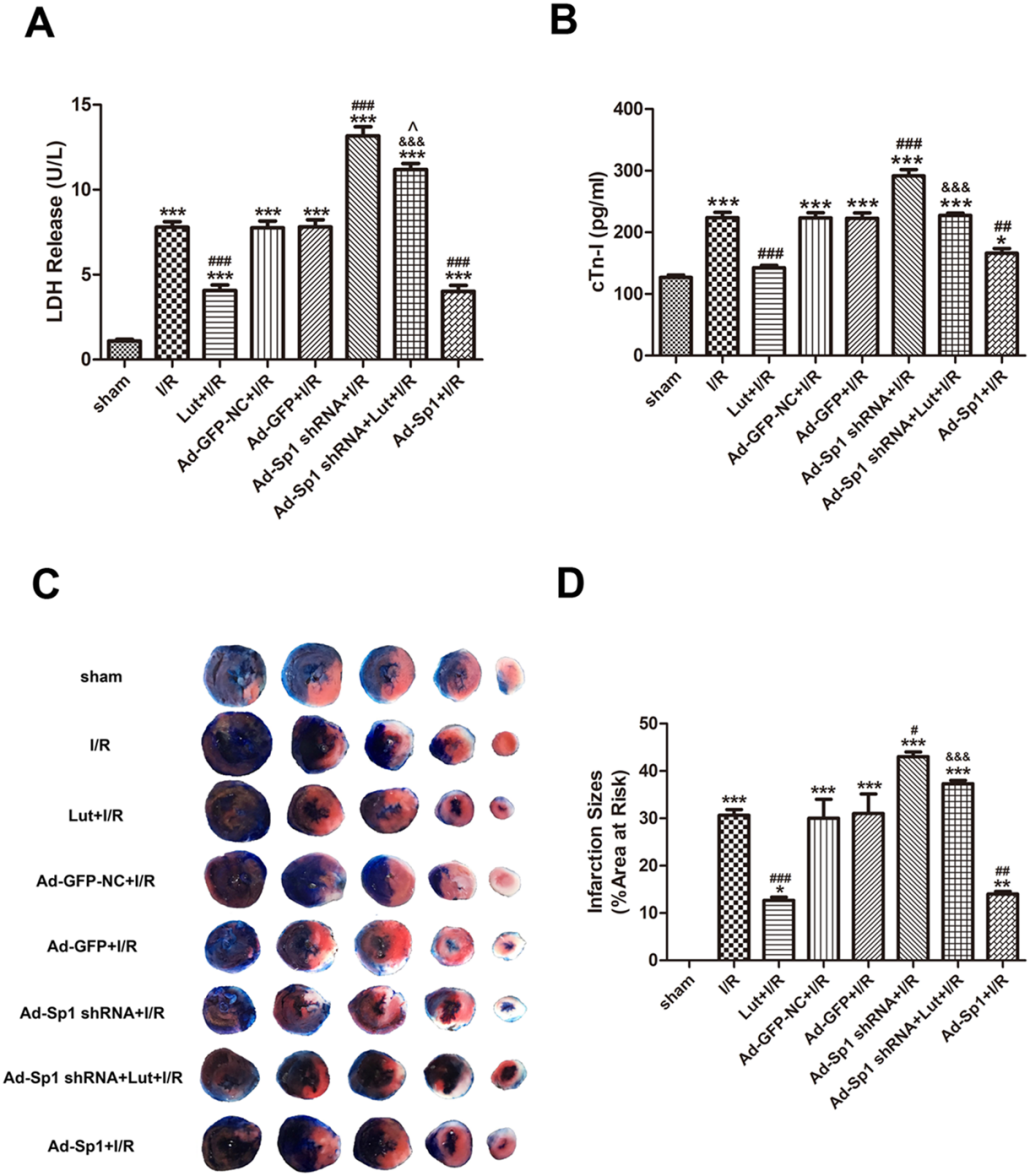
Lut modulates SERCA2a to decrease serum LDH release, reduce cardiac troponin I level and alleviate myocardial infarction via the up-regulation of Sp1. **(A)** LDH release in each treatment group. **(B)** Cardiac troponin I content in each treatment group. **(C)** Representative photographs of Evans blue/TTC-stained heart sections in each treatment group. **(D)** The percentage of infarction size in each treatment group. Data are represented as mean ± SEM (n = 3–5). ^*^P < 0.05, ^**^P < 0.01, ^***^P < 0.001 versus sham; ^#^*P* < 0.05, ^##^*P* < 0.01, ^###^*P* < 0.001 versus I/R; ^&&&^*P* < 0.001 versus Lut + I/R.

As shown (Fig. 4A), an increase in serum LDH release was observed in the I/R group when compared to the sham group (*P* < 0.001). It was partly reversed by Lut administration (*P* < 0.001).

When compared with the I/R group, serum LDH release in the Ad-Sp1 shRNA + I/R group were significantly increased (*P* < 0.001), but levels were decreased in the Ad-Sp1 + I/R group (*P* < 0.001). Serum LDH release in the Ad-Sp1 shRNA + Lut + I/R group was significantly higher than levels in the Lut + I/R group (*P* < 0.001), but levels were lower than those in the Ad-Sp1 shRNA + I/R group (*P* < 0.05) (Fig. 4A).

Furthermore, the trend of serum cardiac troponin I content was consistent with that of the serum LDH release (Fig. 4B).

### Lut modulates SERCA2a to alleviate myocardial infarction via the up-regulation of Sp1

As shown (Supplemental Fig. 1), the percentages of area at risk had no significant differences among all groups. Furthermore, the trend in myocardial infarction size was consistent with that of the serum LDH release (Fig. 4C,D).

### Lut modulates SERCA2a expression to exert anti-apoptotic effects via the upregulation of Sp1

**Figure 5:**
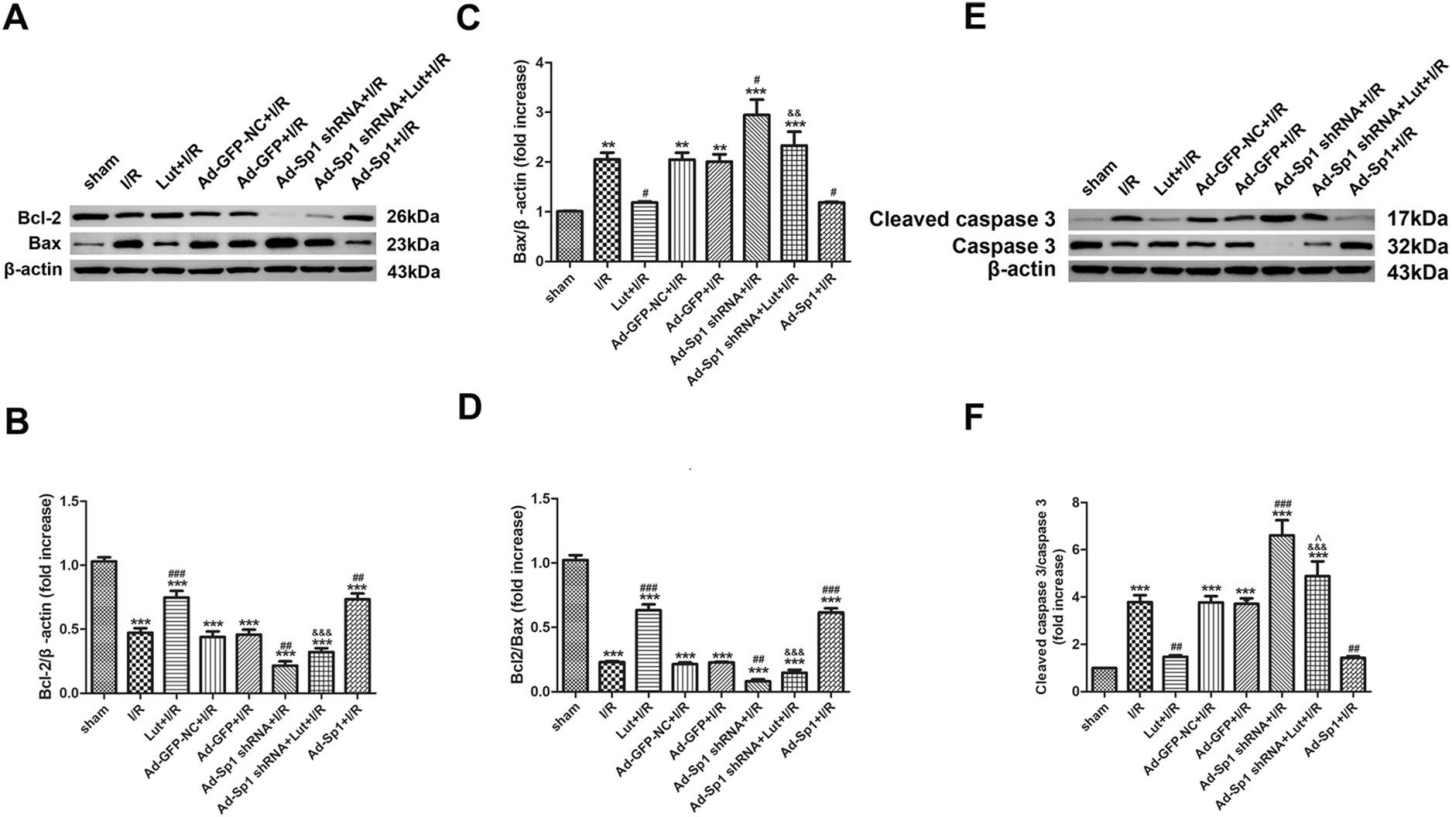
Luteolin modulated SERCA2a via up-regulating Sp1 to inhibit apoptotic proteins expression during myocardial I/R injury. **(A–D)** Expression of Bcl-2 and Bax proteins in each treatment group. **(E, F)** Expression of Cleaved caspase 3 and Caspase 3 proteins in each treatment group. Data are represented as mean ± SEM (n = 5). The full-length blots are presented in Supplemental Fig. 4. ^***^P < 0.001 versus sham; ^##^*P* < 0.01, ^###^*P* < 0.001 versus I/R; ^&&&^*P* < 0.001 versus Lut + I/R; ^^^*P* < 0.05 versus Ad-Sp1 shRNA + I/R.

To investigate apoptosis, the pro-apoptotic expression of Bax and Cleaved caspase 3, anti-apoptotic expression of Bcl-2 and the ratio of Bcl-2/Bax expression were measured. As shown, myocardial I/R injury promoted apoptosis, as the process enhanced the expression of pro-apoptotic markers and reduced the expression of anti-apoptotic proteins. Lut pretreatment significantly reversed the expression of these proteins (Fig. 5A–F).

Pro-apoptotic expression levels were significantly increased when Sp1 was knocked down. However, the pro-apoptotic proteins expression levels were decreased when transfected with a Sp1 overexpression adenovirus. Pro-apoptotic expression levels in the Ad-Sp1 shRNA + Lut + I/R group were significantly higher than those in the Lut + I/R group, but were lower than those in the Ad-Sp1 shRNA + I/R group (Fig. 5A–F).

Moreover, anti-apoptotic expression of Bcl-2 and the ratio of Bcl-2/Bax were completely reversed to the expression level of pro-apoptotic proteins.

### Analysis of chromatin immunoprecipitation (ChIP)

**Figure 6:**
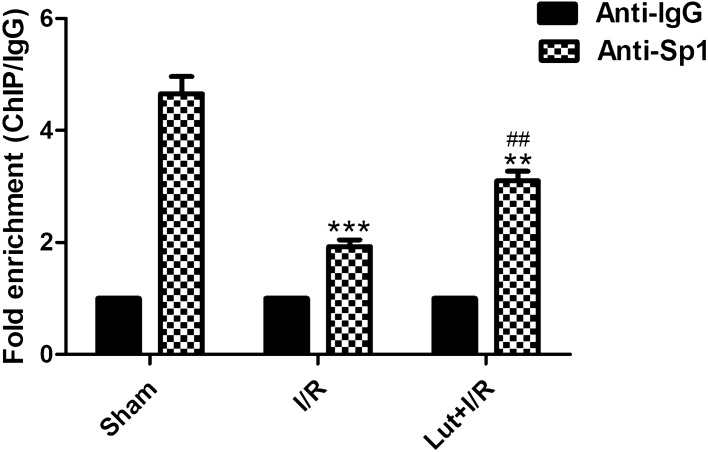
Sp1 transcription factor binding to the SERCA2a promoter region under I/R conditions in the absence or presence of Lut. IgG was used as a negative control. Data are represented as mean ± SEM (n = 3). The picture of agarose gel electrophoresis is presented in Supplemental Fig. 5. ^**^P < 0.01, ^***^P < 0.001 versus sham; ^##^*P* < 0.01 versus I/R.

Sp1 binding activity on the SERCA2a promoter was measured using a ChIP assay. We observed the enrichment for the SERCA2a promoter region under I/R conditions in the absence or presence of Lut versus the negative control IgG (Fig. 6). The obtained results indicate that the enrichment for the SERCA2a promoter region was downregulated in the I/R group, in comparison with the sham group (*P* < 0.001), and the enrichment was partly restored by Lut (*P* < 0.01).

## Discussion

Myocardial I/R injury is one of the most common problems in the clinical treatment of CHD^25^, with calcium overload as the main causative mechanism of such injury. Moreover, it is not only the manifestation, but also the pathological process of myocardial cell death due to reperfusion injury^26–28^.

Calcium overload is closely related to mitochondrial dysfunction and mitochondrial membrane permeability. When subjected to myocardial I/R injury, excessive Ca^2+^ accumulation at the mitochondria leads to calcium overload causing mitochondrial dysfunction and ultimately cell injury and death^29^. Calcium ions in the myocardium are regulated by several factors, e.g. SERCA2a, Na^+^–Ca^2+^ exchangers, L-type calcium channels, ryanodine receptors and other molecules too numerous to mention here^30^. Previous studies have recorded the significance of SERCA2a in this pathology^31,32^. It is now accepted that SERCA2a plays a crucial role in regulating spatial and temporal homeostasis of Ca^2+^ in cardiomyocytes. Our previous reports have shown that SERCA2a expression and activities in isolated Sprague Dawley (SD) rat hearts or myocardial cells, were decreased during myocardial I/R, and similarly, these trends were observed elsewhere in C57/BL6j mice after myocardial reperfusion, over 24 h^14,15^. Although SERCA2a expression in the rat heart was almost unchanged after in vivo reperfusion within 7 days, SERCA2a activity was decreased significantly^33^. The reason for this change is not clear, but it may be explained by the fact that the biological environment of the SD rat model is more complex, e.g., in the in vivo modulation of neuroendocrine factors^34^.

To date, several studies have described multiple Lut pharmacological activities, such as anti-inflammatory, anti-allergic, anti-tumor, anti-atherosclerotic and myocardial protection^35–40^. Lut upregulates the expression of fibroblast growth factor receptor 2 (FGFR2) and leukemia inhibitory factor (LIF) to activate the PI3K/Akt pathway, thereby increasing BAD phosphorylation levels and decreasing Bax;Bcl-2 ratios in cardiomyocytes^41^. Moreover, in diabetic rats, Lut pretreatment reduced the degree of apoptosis and infarct size during myocardial I/R injury^41^. Our previous studies have confirmed that SERCA2a is the main target for Lut pretreatment, in improving myocardial I/R injury^14,15,42^. Lut increased the protein stability of SERCA2a by improving its SUMOylation, which contributed to the upregulation of SERCA2a. It enhanced SERCA2a stability by regulating SERCA2a SUMOylation mainly at lysine 585^15^. However, at the transcriptional field, there has been little discussion on specific Lut mechanisms regulating SERCA2a, requiring further study.

The transcription factor Sp1 regulates the transcription of several genes^43–45^. Takagi et al*.,* used primary human keratinocytes to analyze the structure of the human ATP2A2 promoter. These authors demonstrated that ATP2A2 promoter activity was significantly decreased when the promoter fragments − 550/− 529 bp, − 488/− 472 bp, − 390/− 362 bp or − 42/− 21 bp were deleted. Sp1 binds to the − 550/− 529 bp and − 488/− 472 bp regions of the ATP2A2 promoter. When Sp1 was knocked down, activity of the ATP2A2 promoter and expression levels of ATP2A2 mRNA were significantly decreased^46^. Takizawa et al*.,* suggested that SERCA2 expression was downregulated in the rat model of pressure-overloaded cardiac hypertrophy. The SERCA2 proximal promoter region contains four Sp1 binding sites, and SERCA2 transcriptional activity was changed after mutation of the Sp1 site I (− 196/− 191 bp) and site III (− 118/− 113 bp)^20^. The study also found that carvedilol, a heart medicine, reduced the expression of SERCA2 in a hydrogen peroxide-induced oxidative stress model, and that carvedilol regulated the transcriptional activity of SERCA2 through the Sp1 site III (− 118/− 113 bp) and Sp1 site IV (− 103/− 98 bp)^47^.

The interaction between promoters and transcription factors is one of the most vital processes in modulating gene expression, so it has become a pivotal breakthrough point in the study of disease mechanism. In the research of Hu et al., measurement of sumoylated Sp1 showed that Lut upregulated SERCA2a transcription might relate to the increase of sumoylated Sp1. They found that upregulation of SUMO1 expression was accompanied by an increase in sumoylation of Sp1 in Lut-treated cardiomyocytes. Therefore, they pointed that Lut upregulated SUMO1 expression, increased sumoylation of Sp1 and eventually promoted SERCA2a transcription in heart failure rats^48^. However, during myocardial I/R injury, there has been a dearth of studies on whether Sp1 is involved in the regulation of SERCA2a promoter activity. Moreover, there has been no reliable evidence to suggest whether Lut regulates the transcriptional activity of SERCA2a through Sp1, to alleviate myocardial I/R injury.

To address these problems, adenoviruses were transfected into mice heart to knock down or overexpress Sp1 in an in vivo model*.* Wild type C57/BL6j mice were selected for these studies. We showed that Sp1 and SERCA2a expression were downregulated, and that these data were consistent with the work of Suh et al*.,* in primary cultured renal proximal tubule cells^49,50^. Lut pretreatment reversed the expression of Sp1 and SERCA2a, suggesting that Lut pretreatment may play a role in attenuating myocardial I/R injury, by upregulating the expression of these two key proteins. However, in human breast cancer cell lines BT474 and MCF-7, Dong et al. found that luteolin downregulated Sp1 activity^51^. Furthermore, in EBV-positive epithelial and B cell lines, luteolin inhibited Sp1 luciferase reporter activity suggesting that disruption of Sp1 binding was involved in the inhibitory mechanism^52^. The opposite tendency of luteolin act to regulate the levels of Sp1 may related to the difference of the cell line and tissue investigated. When Sp1 was knocked down by adenovirus transfection, the expression of SERCA2a was significantly decreased. Similarly, SERCA2a could be markedly upregulated after overexpression of Sp1, indicating that at the transcriptional level, Sp1 may promote SERCA2a expression. When subjected to Lut pretreatment and Sp1 knockdown, the expression levels of SERCA2a were significantly lower than Lut pretreatment levels, meaning that the effect of Lut pretreatment was weakened after the expression of Sp1 was significantly reduced, suggesting that Lut could upregulate the expression of SERCA2a through the transcription factor Sp1.

The mRNA expression of SERCA2a in the Lut pretreatment and Sp1 knockdown groups was higher than levels in the Sp1 knockdown group, suggesting that Lut pretreatment not only partially regulates SERCA2a through Sp1, but also upregulates SERCA2a expression in other pathways at the transcriptional level, e.g., Lut pretreatment could modulate the expression of SERCA2a through HIF-1a or microRNA-146 (our unpublished data). When Sp1 was knocked down and myocardial reperfusion performed, LDH release, cardiac troponin I content, myocardial infarction areas and pro-apoptotic protein expression were increased, and the left ventricular function was more serious. Upon overexpression of Sp1, all indices were significantly improved, suggesting a protective effect of Sp1 on myocardial I/R injury in mice. When Sp1 was knocked down after Lut preconditioning, LDH release, cardiac troponin I content, myocardial infarction area and pro-apoptotic protein expression were increased in comparison with the Lut + I/R group, and left ventricular function was greatly deteriorated. The myocardial protective effects of Lut were weakened after Sp1 knockdown, indicating that Lut may attenuate myocardial I/R injury through Sp1.

The ChIP analyses of the interaction between SERCA2a and Sp1 transcription factor showed higher enrichment for the SERCA2a promoter region by Lut treatment compared with the I/R group. Thus, the results implicate that, at the transcriptional level**,** Lut pretreatment was shown to improve SERCA2a expression via the upregulation of Sp1 to attenuate myocardial ischemia/reperfusion injury.

In summary, Lut pretreatment enhanced the transcriptional activity of SERCA2a by upregulating the expression of Sp1, reducing myocardial injury, improving left ventricular function and reducing cardiomyocyte apoptosis. Therefore, Lut played a protective role during myocardial I/R injury in mice. This may be a crucial anti-apoptotic mechanism, thereby improving the contractile function of cardiomyocytes. At present, the mechanism of Lut pretreatment on protecting the myocardium from I/R injury is not fully elucidated. However, these results provide a new research direction and experimental basis for the study of Lut pretreatment, and they lay a foundation for future applications of Lut pretreatment in cardiovascular treatment.

## Materials and methods

### Animals and reagents

Experimental protocols involving animals were approved by the Animal Ethics Committee of the Xuzhou Medical University, Xuzhou, China (permit number: CMCACUC 2016-01-101). Animals were obtained from the Experimental Animal Centre of Xuzhou Medical University, and conformed to the Guide for the Care and Use of Laboratory Animals published by the US National Institutes of Health (NIH Publication No. 85-23, revised 1996) and ARRIVE guidelines. Luteolin (Fluka; purity > 98%; Sigma-Aldrich, Seelze, Germany), after dissolving in dimethyl sulphoxide (DMSO) was diluted in PBS. The concentration of DMSO in the final reagent was less than 0.01%, which has been shown to exert no side-effects on heart tissue^15,53^.

### In vivo I/R injury model of mice hearts

Before subjection to myocardial I/R injury, mice were fasted for 12 h. Animals were placed in a plastic box, subjected to 4% isoflurane, and placed on a heating pad and anesthetized. After fur was removed from the left chest, mice were fixed in the right lateral decubitus position and maintained with 1.0–1.5% isoflurane through a facemask. After sterilization with iodophor (× 3), the surgical site was covered with a sterile sheet.

The myocardial I/R injury procedure was performed in accordance with the method established by our laboratory^54^. When the third and fourth intercostal space was exposed, cardiac impulses were observed through the muscle layer. A 5 mm incision was placed between the two ribs, where cardiac impulses were the most obvious, exposing the internal thoracic cavity. The left anterior descending coronary artery (LAD) was observed below the left auricle. A silk suture, which was threaded underneath the LAD, was tied in a surgical knot, where an uninflated balloon was placed in a surgical knot on the LAD. Finally, the LAD bloodstream was blocked when filling up the surgical knot with the inflated balloon. A change in electrocardiography (ECG) confirmed that the model was successfully established, followed by ST segment of ECG elevated. The balloon was deflated and pulled out after 30 min of ischemia. Subsequently, the ST segment of the ECG was declined. Operative treatment in the sham group involved the passing of the suture thread underneath the left anterior descending branch, without ligation. All subsequent experiments were carried out after 24 h of myocardial reperfusion.

### Lut pretreatment

Before subjection to myocardial I/R injury, the mice used for Lut preconditioning were given an intravenous injection of Lut into the tail vein (15 µg/kg dose). Other groups were administered DMSO diluent for three days. The dose and concentration of DMSO in the Lut solution were the same as those of DMSO injected into the other groups ^15^.

### Adenovirus transfection

The adenoviruses were generated by Hanbio Biotechnology (Shanghai, China) and comprised of replication-deficient adenoviral vectors, encoding an Sp1 interfering sequence with green fluorescent protein (Ad-Sp1 shRNA) or an Sp1 overexpression sequence with green fluorescent protein (Ad-Sp1) or green fluorescent protein (GFP) alone. The Sp1 short hairpin RNA (shRNA) targeting sequence was: 5-AGGACAGACTCAGTATGTGACCAATGTAC-3. After inhaling 4% isoflurane, mice were fixed on the heating pad, in the right lateral decubitus position, and maintained on 1.0–1.5% isoflurane, through a facemask. After sterilization with iodophor (3 ×), the surgical site was covered with a sterile sheet. After opening the thoracic cavity, adenovirus was injected into the myocardium at three adjacent sites. The viral dose was 10^10^ pfu/mL in 10 µL, as recommended by Hanbio Biotechnology. Approximately 72 h later, protein and mRNA expression levels of Sp1 were examined to verify adenovirus transfection efficiency using western blotting and PCR approaches, or the mice were used to establish myocardial I/R injury models^55^.

### Grouping schemes and intervention measurements of each group

The pathogen-free adult male C57BL/6j mice (n = 144), weighing 20–25 g, were randomly divided into eight groups, (1) sham group, (2) I/R group, (3) Lut + I/R group, (4) Ad-GFP-NC + I/R group, (5) Ad-GFP + I/R group, (6) Ad-Sp1 shRNA + I/R group, (7) Ad-Sp1 shRNA + Lut + I/R group and (8) Ad-Sp1 + I/R group, therefore each group contained 12 mice. The pathogen-free adult male C57BL/6j mice (n = 15) were randomly divided into five groups, including the (1) control (con) group, (2) Ad-GFP group, (3) Ad-GFP-NC group, (4) Ad-Sp1 shRNA group and the (5) Ad-Sp1 group. Among them, the con group, Ad-GFP-NC group, Ad-GFP group, Ad-Sp1 shRNA group and Ad-Sp1 group were used to detect the transfection efficiency of the adenovirus. Mice in the con group were wild-type, and were not transfected by adenovirus. The pathogen-free adult male C57BL/6j mice (n = 9) were randomly divided into three groups, including the (1) sham group, (2) I/R group, (3) Lut + I/R group, which were used to accomplish the ChIP assay. Mice in the Ad-GFP group were transfected with a Sp1 overexpression control adenovirus, using three-point injection. Mice in the Ad-GFP-NC group were transfected with a Sp1 knockdown control adenovirus. Mice in the Ad-Sp1 group were transfected with a Sp1 overexpression adenovirus. Mice in the Ad-Sp1 shRNA group were transfected with a Sp1 knockdown adenovirus. The I/R myocardium models were constructed after transfection with the relevant adenovirus and/or pretreated with Lut for three days. Mice in the sham group were threaded the suture underneath the left anterior descending branch, without ligation.

### Measurement of plasma lactate dehydrogenase (LDH) levels

The carotid blood of each mouse was collected when subjected to 24 h reperfusion. The concentration of plasma LDH was assessed according to manufacturer’s instructions (BioVison, Milpitas, CA, USA). The OD_450nm_ values of each group was measured using a microplate reader (Bio-Rad 550, Hercules, CA, USA) and LDH release was calculated according to manufacturer’s instructions.

### Measurement of cardiac troponin-I (cTn I) in serum

Serum cTn I is a specific biomarker for reflecting the myocardial necrosis level after myocardial I/R injury. The serum content of cTn-I was measured by enzyme-linked immunosorbent assay kits (ELISA Kit, Cusabio). Multi-detection microplate reader (Synergy™HT, BioTek, USA) was used for detection. All protocols were carried out in accordance with kit manufacturing recommendations.

### Hemodynamics monitoring

The animals inhaled 1% isoflurane after a 24 h reperfusion. After fixing to the heating pad in the supine position, mice were sterilized with iodophor (3 ×), followed by isolation of the right carotid artery. A heparin blood tube, was sent to the left ventricle of mice from right carotid artery. After stabilization, the heart rate (HR), maximal rate of increase of left ventricular pressure (+ dP/dtmax)*,* maximal rate of decrease of left ventricular pressure (-dP/dtmax)*,* left ventricular systolic pressure (LVSP) and left ventricular end-diastolic pressure (LVEDP) were monitored*. *Finally, tested mice were administered with pentobarbital sodium (150 mg/kg).

### SERCA2a activity measurement

The ultramicro-ATPase assay kit, which comprises an inorganic phosphorus colorimetric method was used to detect SERCA2a activity in cardiac tissue, as per manufacturer’s instructions (Jiancheng Bioengineering Institute, Nanjing, China).

### Myocardial infarct size detection

This metric was measured by Evans blue and 2,3,5-triphenyltetrazolium chloride (TTC, Sigma-Aldrich, St. Louis, MO, USA) staining. Animals inhaled 1% isoflurane after a 24 h reperfusion. The original ligation site of the left anterior descending branch was tied to obstruct blood flow, then the aorta was carefully separated. After clamping the aortic root with a vascular clip, approximately 0.4 mL 2% Evans Blue was injected into the left atrial cavity to ensure the myocardial tissue of the non-left anterior descending branch blood-supply area was adequately stained. 10% potassium chloride was administered at the injection point to ensure the cardiac impulse stopped in the diastolic phase. After this, hearts were excised and frozen for 30 min and the tissue chopped into five slices. The thickness of each slice was approximately 1.2 mm. Each slice was incubated with 1% TTC at 37 °C for 20 min. The color of the infarct area was white. The blue area was referred to as the non-ischemic area, and the red and white area was referred to as the area at risk (AAR). Infarct size was represented as the percentage of area at risk. The area at risk size was presented as the percentage of the total cross-sectional area of the heart.

### RNA extraction and quantitative reverse transcription polymerase chain reaction (qRT-PCR)

Total RNA was extracted from isolated myocardial tissue using TRIzol reagent (Invitrogen, Carlsbad, CA, USA). The mRNA from different samples was analyzed according to a previous protocol^56^. Experimental data was normalized to β-actin and calculated as 2^–ΔΔCT^. ChIP was performed using a Sp1-specific antibody (Cell Signaling Technology no. 9389S) and Simple ChIP reagents (Cell Signaling Technology no. 9002), according to manufacturer’s instructions. qPCR and ChIP primers were as follows:

Sp1: forward: 5′-ACTGTGAATGCTGCTCAACTCTC-3'
  reverse: 5′-CACCTGCTGTCTCATCATGTATC-3'
SERCA2a: forward: 5′ -CGGTGCCTTTGTTGTCTCCA-3'
  reverse: 5′-ACCTGACTTTCGTCGGCTGTGT-3'
β-actin: forward: 5′-GGGAAATCGTGCGTGAC-3'
  reverse: 5′-AGGCTGGAAAAGAGCCT-3'.

### Western blotting

Myocardial samples were lysed to extract protein which was subjected to SDS–polyacrylamide gel electrophoresis, and transferred to polyvinylidene fluoride membranes (Merck Millipore, Billerica, MA, USA). Western blotting was performed according to a previous protocol^57^ using diluted primary antibodies against SERCA2a (1:2,000), Sp1 (1:200), Bax (1:1,000) (Santa Cruz Biotechnology, Santa Cruz, CA, USA), Bcl-2 (1:1,000) (Abcam, Cambridge, UK) and caspase 3 (1:1,000) (Cell Signaling Technology, CST, USA). Anti-mouse-HRP and anti-rabbit-HRP secondary antibodies were purchased from ZSGB-BIO (Beijing, China). Protein bands were visualized using a chemiluminescence detection system (Tanon Imaging System, Tanon, Shanghai, China).

### Statistical analysis

Data are presented as mean ± standard errors of the mean and analyzed by GraphPad Prism 5.0 software. Data between two groups were compared using two-tailed Student's *t*-tests and multiple comparisons utilized one-way ANOVA followed by a Bonferroni post hoc correction test. A P < 0.05 was considered statistically significant.

## Supplementary information

Supplementary Information.
