# Supplementary material for: Luteolin modulates SERCA2a via Sp1 upregulation to attenuate myocardial ischemia/reperfusion injury in mice
Source: Sci Rep. 2020 Sep 21;10:15407. doi: 10.1038/s41598-020-72325-8 (PMC7506543; doi:10.1038/s41598-020-72325-8)
Supplement: Supplementary file 1 — Supplementary Information. [file 41598_2020_72325_MOESM1_ESM.docx]

**Luteolin** **modulates SERCA2a via Sp1 upregulation to attenuate myocardial ischemia/reperfusion injury in mice**

Ya Hu^1^, Chengmeng Zhang^1^, Hong Zhu^2^, Shuai Wang^2^, Yao Zhou^1^, Jiaqi Zhao^1^, Yong Xia^2^**^*^**, Dongye Li^1,2^**^*^**

^1^Institute of Cardiovascular Disease Research, Xuzhou Medical University, 221002, Xuzhou, Jiangsu, PR China
^2^Department of Cardiology, The Affiliated Hospital of Xuzhou Medical University, 221002, Xuzhou, Jiangsu, PR China

***Correspondence to**: Dongye Li or Yong Xia.
Dongye Li, Institute of Cardiovascular Disease Research, Xuzhou Medical University, 84 West Huaihai Road, 221002, Xuzhou, Jiangsu, PR China. Department of Cardiology, The Affiliated Hospital of Xuzhou Medical University, 99 West Huaihai Road, 221002, Xuzhou, Jiangsu, PR China.
Tel: +86-516-85582763;
Fax: +86-516-85582753.
E-mail: [dongyeli@xzhmu.edu.cn](mailto:dongyeli@xzhmu.edu.cn).

Yong Xia, Department of Cardiology, The Affiliated Hospital of Xuzhou Medical University, 99 West Huaihai Road, 221002, Xuzhou, Jiangsu, PR China.

E-mail: [xiayongphd@163.com](mailto:xiayongphd@163.com).

Y. Hu and C. Zhang contributed equally to this work.


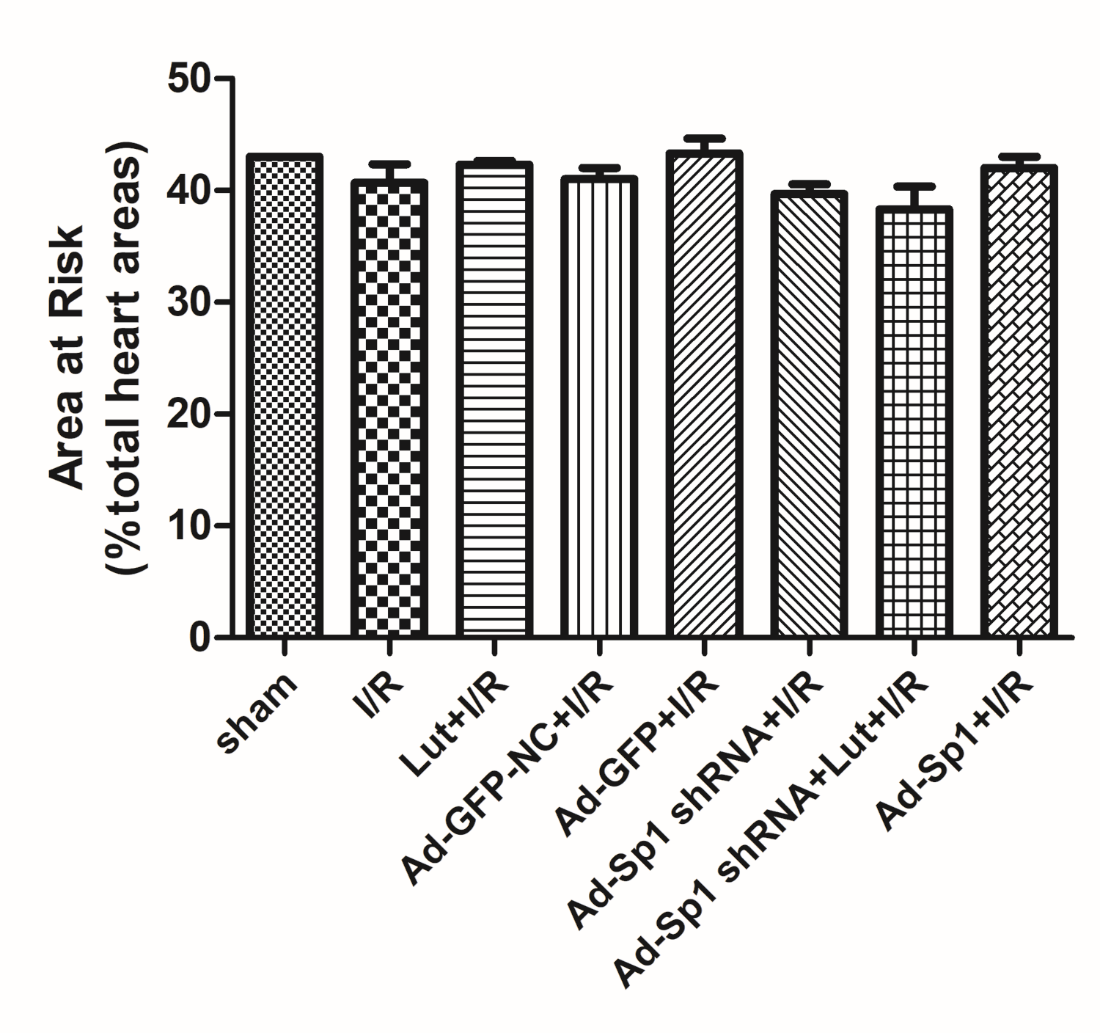


**Supplemental fig.1** The percentage of area at risk in each treatment group.


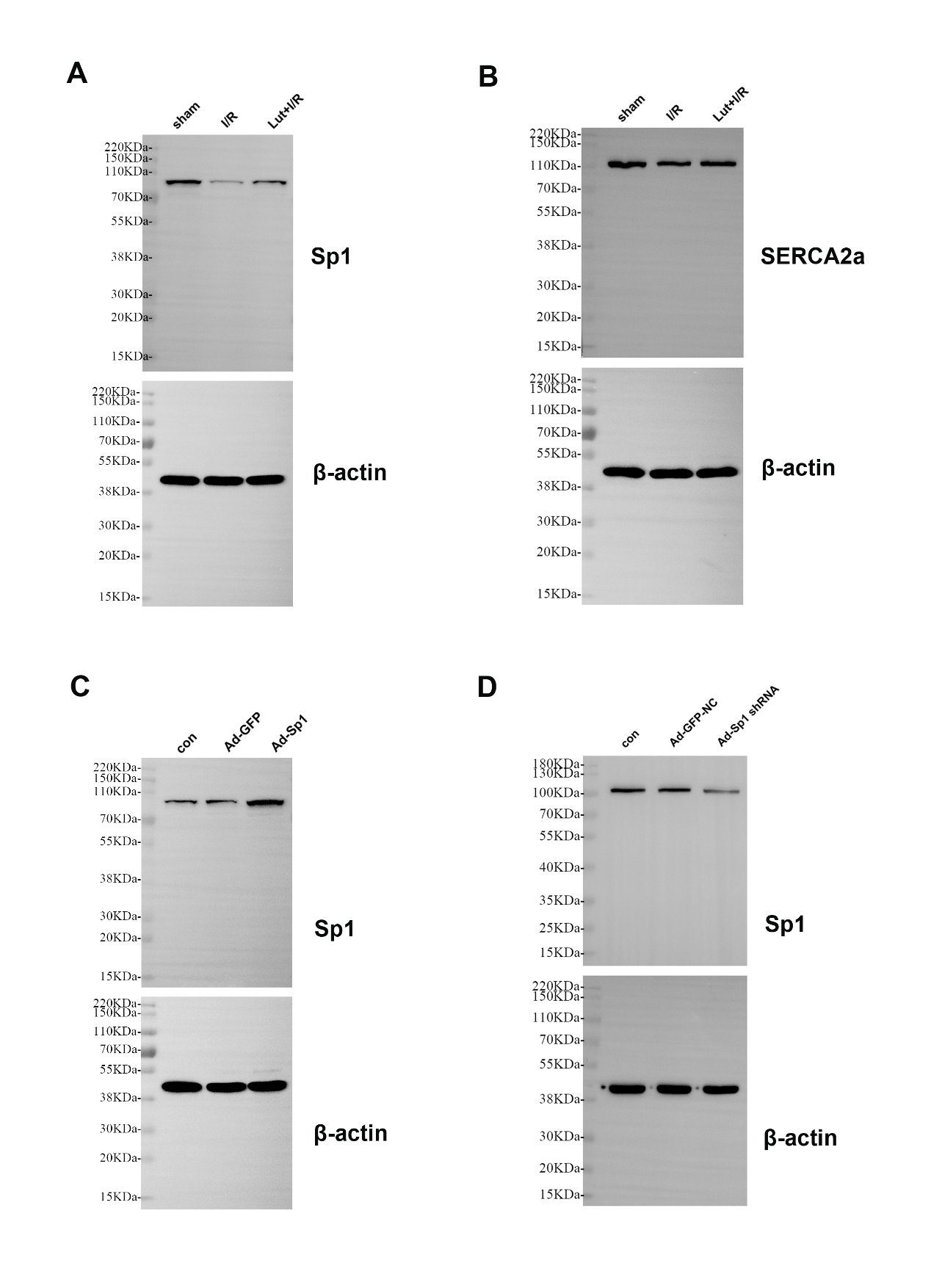


**Supplemental fig.2** The full-length blots of Figure 1.


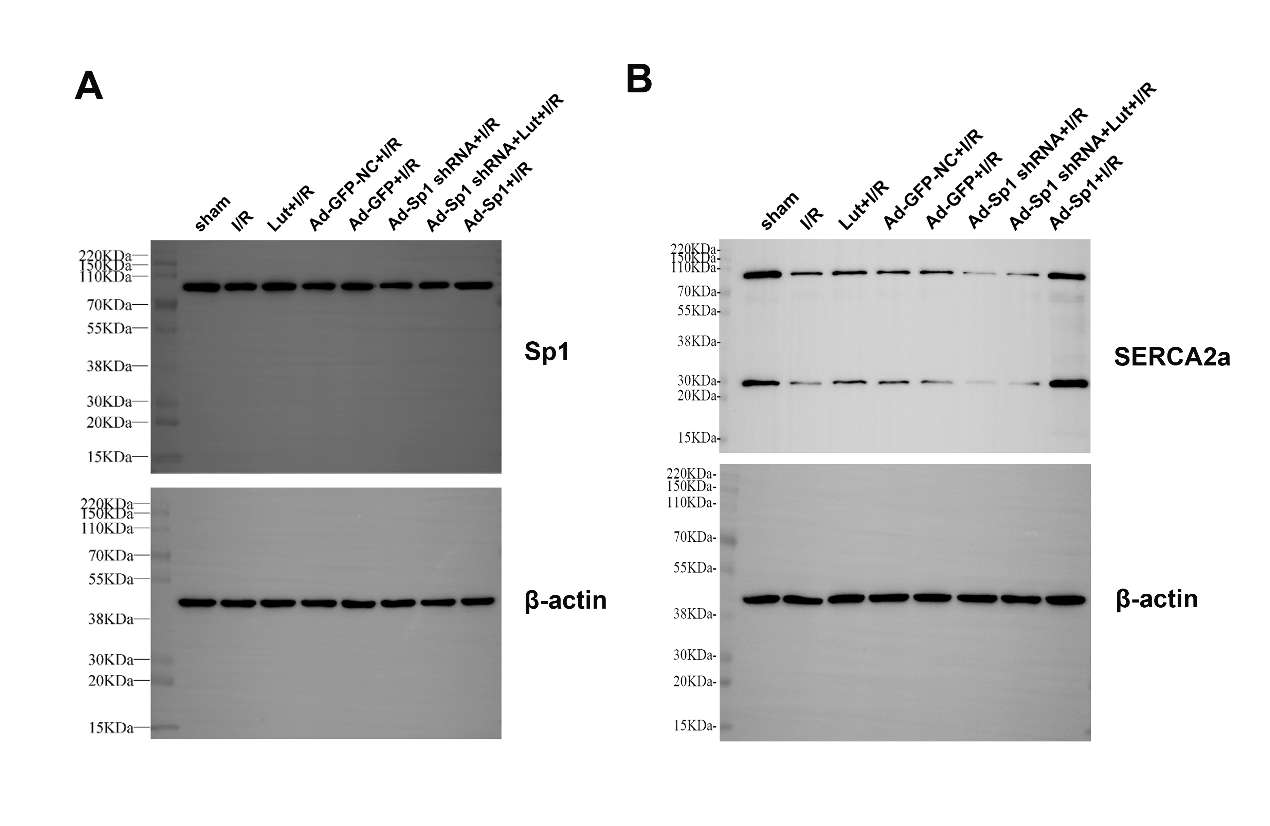


**Supplemental fig.3** The full-length blots of Figure 2.


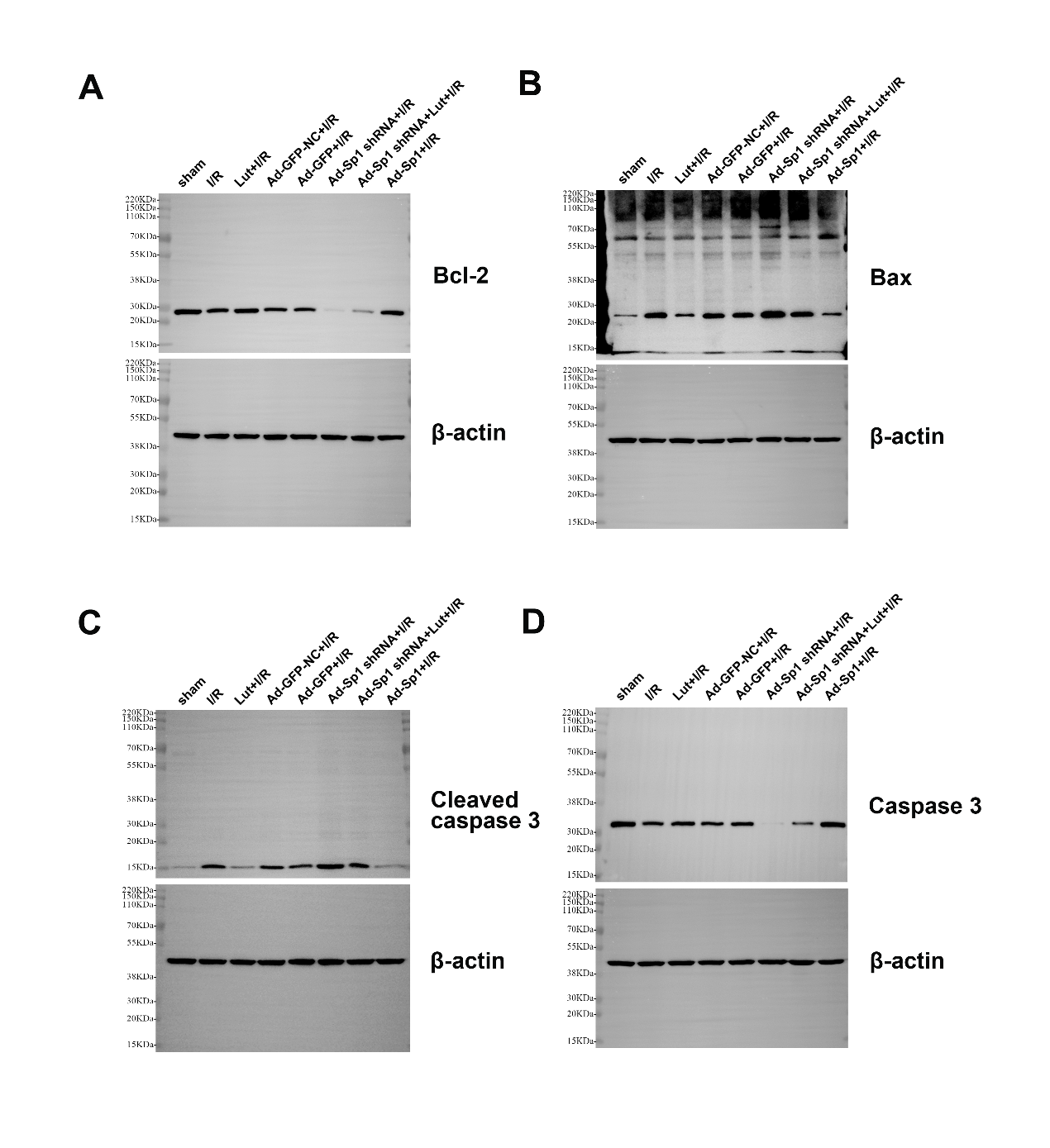


**Supplemental fig.4** The full-length blots of Figure 5.


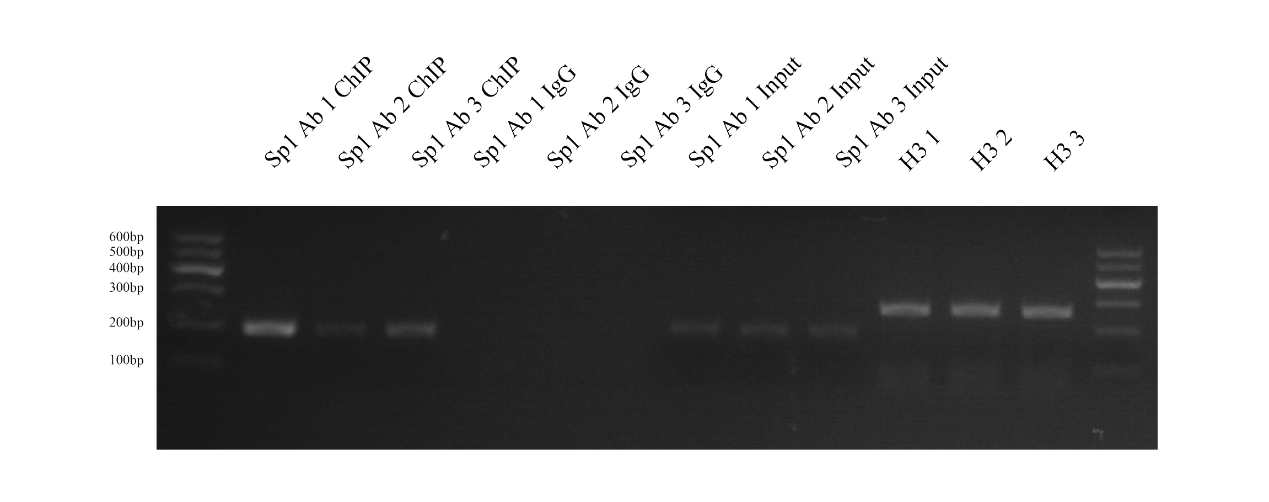


**Supplemental fig.5** The PCR products of ChIP assay were identified by agarose gel electrophoresis.
